# Favipiravir and/or nitazoxanide: a randomized, double-blind, placebo-controlled trial of early therapy in COVID-19 in health workers, their household members, and patients treated at IMSS (FANTAZE)

**DOI:** 10.1186/s13063-022-06533-0

**Published:** 2022-07-22

**Authors:** Tania Smith, Carlos Hoyo-Vadillo, Akosua Agyeman Adom, Liliana Favari-Perozzi, Silke Gastine, Hakim-Moulay Dehbi, Beatriz Villegas-Lara, Eduardo Mateos, Yessica Sara Pérez González, Maria D. Navarro-Gualito, Alejandra S. Cruz-Carbajal, Miguel A. Cortes-Vazquez, Carolina Bekker-Méndez, Charmina Aguirre-Alvarado, Gisela Aguirre-Gil, Lucero Delgado-Pastelin, Andrew Owen, David Lowe, Joseph Standing, Jorge Escobedo

**Affiliations:** 1 CINVESTAV-IPN, Mexico, Mexico; 2 ICH-UCL, London, UK; 3 Clinical Trials Unit, UCL, London, UK; 4 IMSS, Mexico, Mexico; 5 Hakken Enterprise, Cuernavaca, Mexico; 6 University of Liverpool, Liverpool, UK; 7 Division of Infection and Immunity, UCL, London, UK

**Keywords:** COVID-19, Antivirals, Combination therapy, Early treatment, 2×2 design, Favipiravir, Nitazoxanide, Placebo-controlled trial, Protocol, Randomised controlled trial

## Abstract

**Background:**

The 2020 pandemic of SARS-CoV-2 causing COVID-19 disease is an unprecedented global emergency. COVID-19 appears to be a disease with an early phase where the virus replicates, coinciding with the first presentation of symptoms, followed by a later ‘inflammatory’ phase which results in severe disease in some individuals. It is known from other rapidly progressive infections such as sepsis and influenza that early treatment with antimicrobials is associated with a better outcome. The hypothesis is that this holds for COVID-19 and that early antiviral treatment may prevent progression to the later phase of the disease.

**Methods:**

Trial design: Phase IIA randomised, double-blind, 2 × 2 design, placebo-controlled, interventional trial.

Randomisation: Participants will be randomised 1:1 by stratification, with the following factors: gender, obesity, symptomatic or asymptomatic, current smoking status presence or absence of comorbidity, and if the participant has or has not been vaccinated. Blinding: Participants and investigators will both be blinded to treatment allocation (double-blind).

**Discussion:**

We propose to conduct a proof-of-principle placebo-controlled clinical trial of favipiravir plus or minus nitazoxanide in health workers, their household members and patients treated at the Mexican Social Security Institute (IMSS) facilities. Participants with or without symptomatic COVID-19 or who tested positive will be assigned to receive favipiravir plus nitazoxanide or favipiravir plus nitazoxanide placebo. The primary outcome will be the difference in the amount of virus (‘viral load’) in the upper respiratory tract after 5 days of therapy. Secondary outcomes will include hospitalization, major morbidity and mortality, pharmacokinetics, and impact of antiviral therapy on viral genetic mutation rate. If favipiravir with nitazoxanide demonstrates important antiviral effects without significant toxicity, there will be a strong case for a larger trial in people at high risk of hospitalization or intensive care admission, for example older patients and/or those with comorbidities and with early disease.

**Trial registration:**

ClinicalTrials.govNCT04918927. Registered on June 9, 2021.

## Administrative information

Note: the numbers in curly brackets in this protocol refer to SPIRIT checklist item numbers. The order of the items has been modified to group similar items (see http://www.equator-network.org/reporting-guidelines/spirit-2013-statement-defining-standard-protocol-items-for-clinical-trials/).

**Table:** 

Title {1}	Favipiravir and/or nitazoxanide: a randomized, double-blind, 2x2 design, placebo-controlled trial of early therapy in COVID-19 in health workers, their household members, and patients (FANTAZE).
Trial registration {2a and 2b}.	Register at clinicaltrials.gov: NTC04918927 dated June 9th, 2021. https://clinicaltrials.gov/ct2/show/NCT04918927
Protocol version {3}	Version: 1.4, Date: November 12th, 2021.
Funding {4}	Siegfried Rhein S.A. de C.V. is the sponsor of the primary outcome. Other sponsors may be approached for the secondary outcomes.
Author details {5a}	Prof. Joseph Standing, Dr. David Lowe, and Hakin-Moulay Dehbi designed and wrote the original trial protocol based on the FLARE protocol sponsored by UCL and coordinated by CCTU. Dr. Carlos Hoyo-Vadillo, Dr. Liliana Favari Perozzi and M. Sc. Tania Smith translated the original protocol to Spanish and included Mexican applicable legislation. They also contacted and negotiated conditions with the funder of the project: Siegfried Rhein, S.A. de C.V. Dr. Carolina Bekker-Mendez, Dr. Gisela Aguirre-Gil, Ms. C. Lucero Delgado-Pastelin and Dr. Charmina Aguirre-Alvarado designed and validated the q-RT-PCR technique for both nasal swab and stool samples. They also wrote the Biosafety section of this protocol. Dr. Jorge Escobedo de la Peña, Dr. Eduardo Mateos, Dr. Yessica Pérez, MD Maria D. Navarro-Gualito, MD Alejandra S. Cruz-Carvajal MD Miguel A. Corte-Vazquez and M. Sc. Beatriz Viillegas reviewed and pre-approved the protocol and submitted and followed up its authorization by the Trial Steering Committee, the National Committee of Sicentific Investigation (IRB00003566) and the Mexican Regulator: Federal Commission for the Protection against Sanitary Risks (COFEPRIS).
Name and contact information for the trial sponsor {5b}	Contact for Public Queries: Dr. Jorge Escobedo de la Peña, Instituto Mexicano del Seguro Social (IMSS). jorgeep@unam.mx Contact for Scientific Queries: Prof. Joseph F. Standing, University College London (UCL). j.standing@ucl.ac.uk
Role of sponsor {5c}	The role of the study funder (Sigfried Rhein) will be to provide financial and logistic support and will not be involved in the study design, management, collection, analysis, interpretation of data or, report writing. The sponsor (IMSS) will be responsible of the trial management as well as for all actions and activities involving the care of the participants during the trial including but not limited to: selection; recruitment, informed consent obtention, eligibility confirmation, interventions; sample collection, handling and storage prior sample analysis; data collection and, adverse events reports.

## Introduction

### Background and rationale {6a}

In viral infections such as influenza, it is well recognized that early antiviral therapy is associated with improved clinical outcomes. Using drugs with differing modes of action is a fundamental principle of antimicrobial chemotherapy and is used across the spectrum of infectious diseases (for example in HIV, tuberculosis, multi-drug resistant bacteria and invasive fungal disease).

The antiviral drug favipiravir is active against a broad range of viruses and has shown promising results in COVID-19 disease in two small Chinese studies [1, 2]. The drug works similarly to remdesivir (another promising treatment for COVID-19), but unlike remdesivir (which is intravenous) it has a well-established and currently available oral formulation with a good safety profile. Furthermore, both Royal Free Hospital and Great Ormond Street Hospital (London) involved in this trial are amongst the only UK groups with direct clinical experience of this drug, having successfully used favipiravir to treat other viral infections.

The results of the early studies [1, 2] on favipiravir in COVID-19 urgently need to be confirmed or refuted in a high-quality placebo-controlled trial.

Another antiviral with potential activity in COVID-19 is nitazoxanide. Nitazoxanide and its active metabolite, tizoxanide, are active against a variety of bacteria and have also been found to have activity against many DNA and RNA viruses. Nitazoxanide acts as an antiviral at the step after favipiravir in that it inhibits blocks maturation of the viral nucleocapsid N protein and therefore viral assembly [3]. It has also been suggested that nitazoxanide may have non-specific beneficial effects including innate immune system boosting and damping late immune and inflammatory responses [3, 4].

Favipiravir and nitazoxanide have different mechanisms of action so given together are likely to act at least additively, and possibly synergistically, providing a strong rationale for a trial which examines combination therapy.

Health workers are at high risk of acquiring SARS-CoV-2 infection, as already demonstrated in areas which have experienced significant outbreaks. Apart from the risk of severe illness and death in the workers themselves and their household members, this phenomenon also impacts staffing levels in critical systems. There is an urgent need to study subjects who have recently developed symptoms or have recently been tested positive with or without symptoms, and who can be sampled frequently to understand changes in viral load. Conducting a trial including, but not limited to, health workers and their household members can help fulfilling this need. In addition to being at great need for therapeutic interventions, this population provides the ideal cohort to collect detailed trajectory data on early disease and understand how pharmacological interventions may affect this.

The design of the FANTAZE trial is based on the FLARE trial [5] conducted in the UK in 2020–2021.

### Objectives {7}

The primary objective of this trial is to assess whether early antiviral therapy with favipiravir + nitazoxanide is associated with a decrease in viral load compared with favipiravir alone.

Secondary objectives will include hospitalization, major morbidity and mortality, pharmacokinetics, and impact of antiviral therapy on viral genetic mutation rate.

### Trial design {8}

FANTAZE is a phase IIA randomised, double-blind, placebo-controlled, interventional trial.

## Methods: participants, interventions and outcomes

### Study setting {9}

The Mexican Institute of Social Security (IMSS) will be responsible for both the research centres where the trial will be carried out, as well as for the selection of the researchers that will participate in the trial.

The study will be carried out at the Infectious Disease National Medical Centre “La RAZA” Hospital. Participants will be recruited among health workers, their household members and patients that are diagnosed at La Raza or any of the outpatient clinics in its service area (north section of Mexico City and its suburbs).

### Eligibility criteria {10}

Participants will be considered eligible for enrolment in this trial if they fulfil all the inclusion criteria and none of the exclusion criteria as defined below.

#### Participant inclusion criteria

1. Health workers, their household members and patients treated at IMSS facilities with the following:

- Symptoms compatible with COVID-19 disease (fever >37.8°C on at least one occasion and either cough and/ or anosmia) within the first 5 days of symptom onset (date/time of enrolment must be within the first 5 days of symptom onset).
- Or any symptoms compatible with COVID-19 disease (may include, but are not limited to fever, cough, shortness of breath, malaise, myalgia, headache, coryza) and tested positive for SARS-CoV-2 within the first 5 days of symptom onset (date/time of enrolment must be within the first 7 days of symptom onset).
- Or no symptoms but tested positive for SARS-CoV-2 within the last 48 h (date/time of test must be within 48 h of enrolment).

2. Male or female aged 18 years to 70 years old inclusive at screening.
3. Willing and able to take daily nose swab samples.
4. Able to provide full informed consent and willing to comply with trial-related procedure.

#### Participant exclusion criteria

Exclusion criteria are as follows:

1. Known hypersensitivity to any of the active ingredients or excipients in favipiravir, and in nitazoxanide and matched placebo.
2. Chronic liver disease at screening (known cirrhosis of any aetiology, chronic hepatitis (e.g. autoimmune, viral, steatohepatitis), cholangitis or any known elevation of liver aminotransferases with AST or ALT > 3 X ULN).
3. Chronic kidney disease (stage 3 or beyond) at screening: eGFR < 60 ml/min/1.73m^2^.
4. HIV infection, if untreated, detectable viral load or on protease inhibitor therapy.
5. Any clinical condition which the investigator considers would make the participant unsuitable for the trial.
6. Concomitant medications known to interact with favipiravir, and with nitazoxanide and matched placebo, and carry risk of toxicity for the participant.
7. Current severe illness requiring hospitalization.
8. Pregnancy and/ or breastfeeding.
9. Eligible female participants of childbearing potential and male participants with a partner of childbearing potential not willing to use highly effective contraceptive measures during the trial and within the time point specified following the last trial treatment dose.
10. Participants enrolled in any other interventional drug or vaccine trial (co-enrolment in observational studies is acceptable).

* Considering the importance of early treatment of COVID-19 to impact viral load, the absence of chronic liver/kidney disease will be confirmed verbally by the participant during pre-screening and screening/baseline visit. Safety blood samples will be collected at screening/baseline visit (day 1) and test results will be examined as soon as they become available within 24 h.

#### Screening/baseline visit (day 1)

A delegated site staff will visit the participants at their home. After they have been given sufficient time to ask questions and provided written consent, participants will undergo a final assessment for eligibility (including a urine pregnancy test for women of childbearing potential) and will be recruited to the trial. A baseline nose swab sample for virological analysis and baseline blood samples will be taken. A baseline diagnostic nose and throat swab will be taken if the participant has not been tested for COVID-19 yet.

If they are eligible, participants will be randomised and provided with a trial medication kit, symptoms diary, containers for daily nose swab samples collection, two stool collection containers, a thermometer and instructions for taking their medications, collecting samples and recording daily body temperature readings.

Participants will be considered fully randomised once they take their first trial medication dose, witnessed by the delegated site staff. Arrangements will be made for their next trial visits.

If participants are deemed ineligible following one of the above assessments, data will still be collected on all assessments listed above apart from swab, nose swab and bloods.

The reason(s) for ineligibility will be explained to participants and any questions they have will be answered. They will be thanked for their participation and any relevant information from this will be recorded on the CRF.

If participants are deemed ineligible following results from safety bloods collected at screening/baseline visit, a delegated site research staff will contact the participant (within 6 h of results receipt) to explain the reason(s) for ineligibility and instruct them to interrupt the therapy immediately. The research staff will enquire on any adverse reactions and side effects that the participant might have experienced since starting the trial medications and record details on the screening/baseline CRF. In order to ensure the participant’s safety, arrangements for immediate collection of the medication kit from the participant’s home will be made (within 24 h), and a pills count will be performed on receipt of the medication kit at site.

### Who will take informed consent? {26a}

Written informed consent to enter and be randomised into the trial must be obtained from participants by an investigator (a doctor or a qualified research nurse), after explanation of the aims, methods, benefits, and potential hazards of the trial and before any trial-specific procedures are performed or any samples are taken for the trial.

### Additional consent provisions for collection and use of participant data and biological specimens {26b}

Consent provisions for collection and use of participant data and biological specimens are accounted for in the informed consent form approved by the National Committee of Scientific Investigation (IRB00003566) and the Mexican Regulator COFEPRIS.

## Interventions

### Explanation for the choice of comparators {6b}

#### Combination of favipiravir and nitazoxanide

Favipiravir and nitazoxanide have different mechanisms of action so given together are likely to act at least additively.

The preliminary results seen for favipiravir, and the promising in vitro activity of nitazoxanide, urgently need to be confirmed (or refuted) in a well-conducted, placebo-controlled trial. Therefore, there is a strong rationale for choosing to trial early oral antiviral therapy with favipiravir and to investigate its combination with nitazoxanide.

### Intervention description {11a}

Favipiravir 200 mg tablets will be provided free of charge by Strides Pharma Science Limited.

On day 1, 9 tablets (1800 mg) will be taken twice separated by at least 6 h (e.g. if the first dose is at 5 pm, the second dose should be at 11 pm), followed by 2 tablets (400 mg) four (4) times daily from day 2 to day 7.

Nitazoxanide tablets as well as nitazoxanide placebo tablets will be sourced free of charge by Siegfried Rhein, S.A de C.V.

On day 1, 2 tablets (1000 mg) will be taken twice separated by at least 6 h (e.g. if the first dose is at 5 pm, the second dose should be at 11 pm), followed by 1 tablet (500 mg) four (4) times daily from Day 2 to Day 7.

Treatment dosing regimen is summarised in Table 1 below:

**Table 1:** Treatment dosing regimen

Arm	Total daily dosage	Regimen	Total No of tablets daily
**FAVI active + nitazoxanide active**	**Day 1:** FAVI = **3600mg**	**FAVI**: 1st dose: 1800mg (9 tablets) 2nd dose: 1800mg (9 tablets)	**Day 1:** FAVI = 18 tablets
**OR**			
**FAVI active + nitazoxanide placebo**	**PLUS**	**PLUS**	**PLUS**
	NITA = **1000 mg**	**LPV/r:** 1st dose: 1000mg (2 tablets) 2nd dose: 1000mg (2 tablets)	NITA = 4 tablets
	**Day 2 to day 7:** FAVI = **1600mg**	**FAVI**: 1st dose: 400mg (2 tablets) 2nd dose: 400mg (2 tablets) 3rd dose: 400mg (2 tablets) 4th dose: 400mg (2 tablets)	**Day 2 to Day 7:** FAVI = 8 tablets
	**PLUS**	**PLUS**	**PLUS**
	NITA = 5**00mg**	**NITA:** 1st dose: 500mg (1 tablet) 2nd dose: 500mg (1 tablet) 3rd dose: 500mg (1 tablet) 4th dose: 500mg (1 tablet)	NITA = 4 tablets

### Criteria for discontinuing or modifying allocated interventions {11b}

In consenting to the trial, participants are consenting to trial treatments, trial follow-up and data collection. However, an individual participant may stop treatment early or be stopped early for any of the following reasons:

- Unacceptable treatment toxicity or adverse event.
- Inter-current illness that prevents further treatment.
- Any change in the participant’s condition that in the clinician’s opinion justifies the discontinuation of treatment.
- Participant no longer wants to continue taking the trial medications.

### Strategies to improve adherence to interventions {11c}

Every attempt to contact participants who do not adhere to the trial or who terminate participation prematurely will be made. This will include a telephone call on Day 5 from delegated members of the research team. Participants will complete diaries and tablet counts at the end of the intervention period. Reasons for non-adherence or non-retention will be documented.

### Relevant concomitant care permitted or prohibited during the trial {11d}

All medications (except those that are contraindicated according to the nitazoxanide SPC and favipiravir IB) which the trial investigator responsible for the participant’s care feels are clinically appropriate, are permitted in the trial.

If a participant is hospitalised during the trial period they will be encouraged where possible to continue taking trial medication, taking nose swab samples, and recording temperature, but any urgent medical treatment required will be permitted.

### Provisions for post-trial care {30}

No arrangements will be in place for trial treatment after the planned treatment phase. Participants will be cared for as per standard IMSS care should they require further treatment or hospitalisation for their COVID-19 symptoms. This will be made clear in the informed consent form.

### Outcomes {12}

The primary outcome is the upper respiratory tract viral load at day 5.

Method of measurement: quantitative polymerase chain reaction (PCR) performed on nose swab samples.

The secondary outcomes are the following:

- Percentage of participants with undetectable upper respiratory tract viral load after 5 days of therapy.

Method of measurement: quantitative polymerase chain reaction (PCR) performed on nose swab samples.

- Proportion of participants with undetectable stool viral load after 7 days of therapy and 14 days post-randomisation.

Method of measurement: PCR performed on stool samples.

- Rate of decrease in upper respiratory tract viral load during 7 days of therapy.

Method of measurement: PCR performed on daily nose swab samples.

- Duration of fever following commencement of medication.

Methods of measurement: daily body temperature records between Day 1 and Day 7 post- randomization.

- Proportion of participants with hepatotoxicity after 7 days of therapy and 14 days post-randomisation.

Method of measurement: standard diagnostic laboratory assays for liver transaminases, alkaline phosphatase and bilirubin.

- Proportion of participants with other medication-related toxicity after 7 days of therapy and 14 days post-randomisation.

Methods of measurement: determination of medication-related adverse events by investigators.

- Proportion of participants admitted to hospital with COVID-19-related illness.

Methods of measurement: participant self-report, review of hospital records and discharge summaries.

- Proportion of participants admitted to ICU with COVID-19-related illness.

Methods of measurement: participant self-report, review of hospital records and discharge summaries.

- Proportion of participants who have died with COVID-19-related illness.

Methods of measurement: next of kin report, review of hospital records and discharge summaries.

- Pharmacokinetic and pharmacodynamic analysis of favipiravir and tizoxanide.

Method of measurement: assay of favipiravir and tizoxanide levels in plasma at day 7 of therapy. All participants from each arm will provide a pre-dose trough sample and a post-dose (30 to 60 min) sample on Day 7 of therapy. A nonlinear mixed effects model will be fitted jointly to favipiravir and tizoxanide pharmacokinetic and viral load (pharmacodynamic) data.

The model will estimate the following primary PK parameters:

PK: Clearance (CL), volume of distribution (V), absorption rate constant (Ka) from which the following secondary parameters will be derived:

- Maximum concentration (Cmax),
- Time to maximum concentration (Tmax), elimination rate constant (Ke),
- Area under the curve extrapolated to infinity (AUC (0-inf)).
- The model will also estimate the following pharmacodynamic parameters: Rate of viral load decline (delta), and
- Maximum increase in viral load under drug treatment (Emax), Concentration to achieve half the maximum possible effect (EC50).
  - Exploratory: proportion of participants with deleterious or resistance-conferring mutations in SARS-CoV-2.

Method of measurement: deep sequencing of virus and bioinformatic analysis.

### Participant timeline {13}

The participant timeline is shown in Table 2.

**Table 2:**
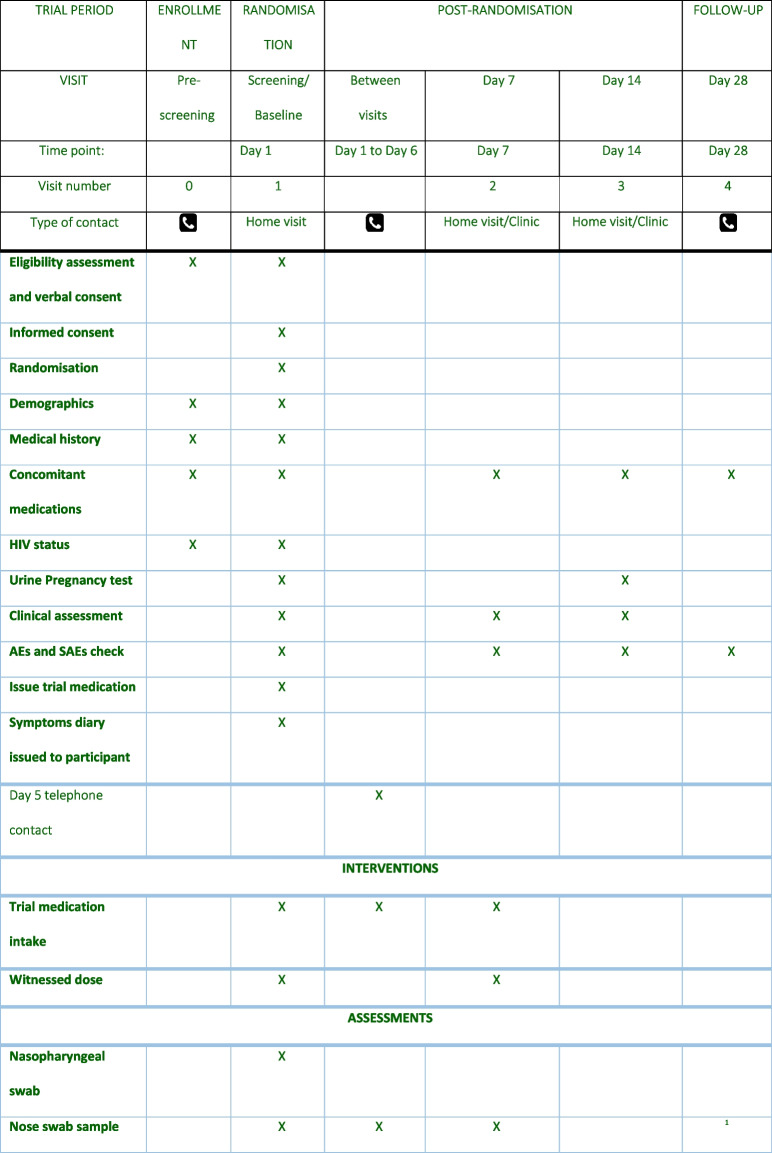
Participant timeline

### Sample size {14}

We have conducted extensive power calculation and power simulation exercises for the proposed design [6]. In the proposed design 120 participants (108 evaluable participants, which implies a 10% allowance for attrition and other methodological challenges) will be recruited, so that half of the participants receive both favipiravir/nitazoxanide treatment, and the other half receive favipiravir/nitazoxanide placebo treatment. We hypothesised that around a 1 log10 difference in viral load by day 5 would be clinically significant.

### Recruitment {15}

The trial will be advertised at each site and the participant identification centre and health workers provided with the trial team’s contact details. Occupational Health teams at each participating centre will be provided with the trial information which they can provide to health workers who consult them. Potential participants will also be approached after research on databases. 120 participants will be recruited in total (60 participants in each arm) over an overall period of 6 months.

Should recruitment targets be slower than anticipated other clinics, hospitals, and diagnostic laboratories in México City and its suburban area will be approached to enroll participants, which will be done centrally by the trial team in any case.

Recruitment will be monitored regularly to identify any barriers to recruitment. Reporting will be conducted on a regular basis over the trial set-up and recruitment period to the Trial Management Group (TMG), and trial oversight committees. Remedial action will be put in place as soon as any concerns have been raised.

## Assignment of interventions: allocation

### Sequence generation {16a}

Participants will be randomised 1:1 using a stratification process, with the following factors: study site, age (≤ 55 vs > 55 years old), gender, obesity (BMI <30 vs ≥30), symptomatic or asymptomatic, current smoking status (Yes = current smoker, No = ex-smoker, never smoker), and the presence or absence of comorbidity (defined as diabetes, hypertension, ischaemic heart disease (including previous myocardial infarction), other heart diseases (arrhythmia and valvular heart disease), asthma, COPD, other chronic respiratory diseases) and, if the participant has or not been vaccinated. These data will be collected at the pre-screening telephone contact and will be used for the purposes of registration and IMP kit pre-allocation.

Considering the importance of starting therapy immediately upon entering this trial, IMP kit codes will be pre-allocated to the participants ahead of the screening/baseline visit on day 1.

The Trial Pharmacologist at CINVESTAV will generate a sequence of unique identifiers for every active/placebo IMP kit. A copy of the sequence will be held securely at UCL and at CINVESTAV.

### Concealment mechanism {16b}

The IMP kit codes will be captured onto RedCap ensuring that the trial team and participants remain blind to treatment allocation.

### Implementation {16c}

Randomisation will be done through the RedCap database using stratification in which patients will first be grouped into strata according to the following factors: age, gender, obesity, symptomatic or asymptomatic, smoking, and vaccination status. Within each stratum, patients will be randomly assigned to one of the two arms of the study.

Following the pre-screening telephone contact before screening/baseline visit, the clinical investigator or delegate will enter the participant’s initials, and all the factors stated above. and confirm participant eligibility on the RedCap secure online system and allocate the appropriate PIN to the patient.

Delegated staff at participating sites will be provided with a secure login to the RedCap website, according to their role in the trial. The randomisation result will be shown directly online as a unique IMP kit code, with an email confirmation sent to the user and to the trial team. The investigator will provide details of the allocated unique kit code assigned to the participant on the prescription. Delegated staff will collect the allocated IMP kit from pharmacy on behalf of the participant ahead of the screening/baseline visit.

Randomisation will be considered complete after the participant has signed the consent form and ingests their first dose of trial medication during screening/baseline visit. Participants who withdraw before taking their first dose will be considered withdrawn prior to randomization.

## Assignment of interventions: blinding

### Who will be blinded {17a}

Participants and investigators will both be blinded to treatment allocation (double-blinded). All IMP will be packaged and labelled to maintain blinding, and blinded IMP kit codes will be used to identify the medication. IMP kit codes will be linked to the PIN allocated to the participant at randomisation.

### Procedure for unblinding if needed {17b}

A member of the CINVESTAV team will unblind the participant’s trial treatment allocation when:

- Emergency unblinding is deemed necessary for appropriate clinical management.
- The participant reports overdosing on study medication and is hospitalised as a result.
- When a SUSAR needs to be submitted to the regulatory agencies within pre-specified timelines.

## Data collection and management

### Plans for assessment and collection of outcomes {18a}

Each participant will be given a unique trial Participant Identification Number (PIN). Data will be collected at the time points indicated in the Participants Timeline (See item {13}).

### Plans to promote participant retention and complete follow-up {18b}

Participants will be in regular contact by phone and text message during the trial duration. They will receive a telephone call on day 5 to schedule their next visit, provide instructions for dose intake on day 7, and verify the nose swab and temperature daily collection as well as the intake of IMP and log registries. The research member will inquire about any clinical events that may require further clinical assessment.

Participants will have two follow-up visits on day 7 and day 14 and will be assessed by the trial team, undergo blood tests for toxicity and pharmacokinetic assessment (on day 7 only), and provide stool samples. These visits can be done in clinic if the participants have completed their isolation period.

Participants will have a telephone follow-up 3 weeks after their last day of treatment (day 7). The research team will enquire about any relevant clinical events that may require further clinical assessment and collect further information through a questionnaire.

### Data management {19}

Data will be entered onto paper Case Report Forms (CRFs) or directly onto the RedCap database. Staff will receive training on data collection. Paper CRFs will be placed in clear plastic wallets which allow reading both sides of the CRF without opening the wallets. The plastic wallets will be decontaminated before leaving the participant's residence as per infection control guidance.

Identification logs, screening and enrolment logs will be kept at the trial site in a locked cabinet within a secured room.

Research team members across all participating sites will receive training on the FANTAZE protocol and on the data entry in the approved FANTAZE database.

All data will be handled in accordance with the Federal Law on Protection of Personal Data Held by Individuals 2010, the Data Protection Act 2018 and the GDPR 2016/679 including all further updates.

### Confidentiality {27}

Adequate measures will be in place to ensure all participant data collected are secure. CRFs will record the participant initials and month/year of birth but not their name. The only link between the PIN and the participant’s name will be on the screening log kept at site and accessed only by the site trial team.

Data will be recorded on the CRFs and entered onto the FANTAZE custom-designed database under this identification number. The database will be password protected and only accessible to members of the FANTAZE trial team at UCL, trained and authorised site staff, and external regulators if requested. The servers are protected by firewalls and are patched and maintained according to best practice.

The physical location of the servers is protected by security door access.

Blood samples will be labelled with the FANTAZE participant identification number ensuring the pseudonymity of the participants who have provided the samples. At the laboratories, brief clinical details will be stored including age and sex. Blood results will be stored on a web-based, secure confidential database, including after completion of the FANTAZE trial.

### Plans for collection, laboratory evaluation and storage of biological specimens for genetic or molecular analysis in this trial/future use {33}

#### Pharmacokinetic sampling

Sample timing: Samples will be drawn for pharmacokinetics on day 7 (Table 3). Participants will be telephoned on Day 5 by the trial site team to receive instructions and arrange the day 7 visit time. The participant will take the medication as instructed by the trial member. The participant will take the dose before the visit and record the time to the nearest minute. The next dose should be taken during the study visit. Before taking the next dose of IMP, a blood sample will be drawn, and time to the nearest minute will be recorded. Once the predose sample has been taken, the participant will take the next dose and record the time to the nearest minute. Two more blood samples will be drawn 30 and 60 minutes after taking the medication. Time to the nearest minute will be recorded on each sample. All samples will be identified with the participant´s PIN, date, and time to the nearest minute. Processing of samples

- Safety bloods (full blood count, urea and electrolytes, liver function tests and uric acid)—sent to the Medical Research Unit in Immunology and Infectology at the National Medical Centre Hospital of Infectology “La Raza” for standard assays.
- Serum for storage—sent to the Medical Research Unit in Immunology and Infectology at the National Medical Centre Hospital of Infectology “La Raza” and processed by the laboratory staff: sample centrifuged and supernatant stored at −70 °C.
- Diagnostic nasopharyngeal swab—sent to the Medical Research Unit in Immunology and Infectology at the National Medical Centre Hospital of Infectology “La Raza” for standard PCR assay for SARS-CoV-2.
- Nose swab and stool samples for viral load measurement and viral sequencing will be sent to the Medical Research Unit in Immunology and Infectology at the National Medical Centre Hospital of Infectology “La Raza” for extraction and storage. Nose swab samples will be kept in a transport medium and stored at 5°C for up to three weeks prior to analysis. Stool samples will be extracted and kept at −70°C prior to analysis.
- Nose swab and stool sample viral load measurements will be done at Hakken Enterprise Laboratories.
- Nose swab and stool samples viral sequencing will be done at UCL.

**Table 3:** Laboratory samples to be collected during the trial

Sample	Day 1	Days 2–6	Day 7	Day 14
Blood samples	• Full blood count (3mL) • Urea and electrolytes, liver function tests, uric acid (7mL) • Serum for storage (10mL) Plasma base line pharmacokinetic samples (1X2 mL)		• Full blood count (3mL) • Urea and electrolytes, liver function tests, uric acid (7mL) • Serum for storage (10mL) • Plasma Pharmacokinetic samples (3×2mL)	• Full blood count (3mL) • Urea and electrolytes, liver function tests, uric acid (7mL) • Serum for storage (10mL)
Nasopharyngeal swab (*only if COVID-19 testing not done yet)*	• Diagnostic sample			
Nose swab	Sample for viral load measurement/viral sequencing	Sample for viral load measurement/viral sequencing taken daily [self- sampled]	Sample for viral load measurement/viral sequencing	
Stool samples			Sample for viral load measurement/viral sequencing	Sample for viral load measurement/viral sequencing

## Statistical methods

### Statistical methods for primary and secondary outcomes {20a}

Summary of baseline characteristics, by trial arms, will be by frequency and percentage for categorical variables, and for continuous variables by mean and standard deviation (or median and inter-quartile range for non-normally distributed data).

The primary endpoint will be analysed using a linear mixed-effect model, including the main effects (each experimental treatment) as factors, and the interaction between them. Baseline values of viral load will be included for each subject and, linked within subjects using random intercept terms.

An analysis of covariance (ANCOVA) will also be performed: the ANCOVA model will use the viral load on Day 5 as the dependent variable and include the baseline value as independent variable.

Secondary endpoints include binary endpoints and continuous endpoints (note that the pharmacokinetic analysis of favipiravir and nitazoxanide is described in a separate section below).

For binary endpoints, a logistic regression will be used. The adjustment strategy will be the same as the primary endpoint.

The comparison of duration of fever following commencement of medication between trial arms will be by t-test, or non-parametric equivalent depending on the distribution of the data. Regression models will also be used to perform analyses adjusted for site (as random effect.).

### Interim analyses {21b}

There will be no interim analyses. Monitoring of the safety of the trial will be undertaken by the IDMC which will have untrammeled access to the trial data, and whose work is governed by a separate charter.

### Methods for additional analyses (e.g. subgroup analyses) {20b}

#### Pharmacometric analysis plan

Population PKPD modelling and dosing simulations will be undertaken with non-linear mixed-effects modelling. Viral load with time will be modelled with a viral dynamic model, and the influence of favipiravir and nitazoxanide included to estimate an in vivo EC50.

Plasma concentrations will be sent from the bioanalytical laboratory on a spreadsheet and dosing history and relevant covariate information extracted from the clinical database. These data will be imported into R (version 3.4 or above) and merged for exploratory analysis and formatted for subsequent modelling using NONMEM version 7.4 or above (Globomax, USA) and or nlmixr version 1.0 of above.

Model selection criteria will include (i) successful stratification, (ii) standard error of estimates, (iii) number of significant digits, (iv) termination of the covariance step and (v) correlation between model parameters. Goodness of fit will be assessed by graphical methods, including population and individual predicted vs. observed concentrations, conditional weighted residuals vs. observed concentrations and time, correlation matrix for fixed vs. random effects, correlation matrix between parameters and covariates and normalised predictive distribution error (NPDE). Comparison of hierarchical models will be based on the likelihood ratio test. A superior model will be also expected to reduce inter-subject variance terms and/or residual error terms. Standard error of the parameter estimates will be approximated using of the asymptotic covariance matrix.

#### Additional analyses

Viral sequencing will be undertaken according to established protocols and analysis of mutagenesis, or the appearance of potentially deleterious mutations will be interrogated as previously described for other RNA viruses.

### Methods in analysis to handle protocol non-adherence and any statistical methods to handle missing data {20c}

The primary analysis will be on the intention-to-treat (ITT) population. There will be no imputation for missing data for any of the study outcomes.

A modified ITT analysis will be performed that will only include participants who have a confirmed diagnosis at baseline of SARS-CoV-2 infection (diagnostic swab and nose swab samples collected at Baseline).

### Plans to give access to the full protocol, participant-level data and statistical code {31c}

The PI, CPM, Trial Manager, Data Manager, Statistician, and Trial Management Team will have full access to the trial data.

#### Reproducible Research

Requests for access to trial data will be considered, and approved in writing where appropriate, after formal application to the TSC.

The full protocol will be available through publication in a peer-reviewed journal.

## Oversight and monitoring

### Composition of the coordinating Centre and trial steering committee {5d}

#### Trial team

The Trial Team (TT) will be set up to assist with developing the design, coordination, and day-to-day operational issues in the management of the trial, including budget management.

#### Trial management group

A Trial Management Group (TMG) will be set up to assist with developing the design, coordination, and strategic management of the trial.

#### Independent trial steering committee

The Independent Trial Steering Committee (TSC) is the independent group responsible for oversight of the trial to safeguard the interests of trial participants. The TSC provides advice to the CI, the funder, and the sponsor on all aspects of the trial.

### Composition of the data monitoring committee, its role and reporting structure {21a}

An Independent Data Monitoring Committee (IDMC) will be appointed for the trial, the IDMC will review unblinded data and make recommendations to the Trial Steering Committee.

### Adverse event reporting and harms {22}

#### Data monitoring for harm

Descriptive statistics will be used to compare rates of adverse events between treatment arms. No additional formal monitoring will be performed except for those required by the IDMC.

All non-serious AEs and ARs, whether expected or not, should be recorded in the participant Adverse Event Log and reported to UCL within 14 days. SAEs and SARs should be notified to IMSS and the Pharmacovigilance Centre immediately after the investigator becomes aware of the event (in no circumstance should this notification take longer than 24 h).

#### Seriousness assessment

When an AE or AR occurs, the investigator responsible for the care of the participant must first assess whether or not the event is serious. If the event is Declassified as ‘serious’ then an SAE form must be completed and IMSS (or delegated body) notified immediately (no longer than 24 h).

#### Severity or grading of adverse events

The severity of all AEs and/or ARs (serious and non-serious) in this trial should be graded using the toxicity gradings in Common Terminology Criteria for Adverse Events (CTCAE) v5.0 [7] , as well in the NOM-220-SSA1-2016 [8].

The investigator must assess the causality of all serious events or reactions in relation to the trial therapy. Any events that may be attributed to treatment should be reported to the Pharmacovigilance Centre according to NOM-220-SSA1-2016 [8].

IMSS is responsible for the reporting of SUSARs and other SARs to the Pharmacovigilance Centre and the corresponding EC. Fatal and life-threatening SUSARs must be reported to the competent authorities within 7 days of UCL becoming aware of the event; other SUSARs must be reported within 15 days.

IMSS will keep investigators informed of any safety issues that arise during the trial.

The trial manager or delegate at IMSS will submit Development Safety Update Reports (DSURs) to competent authorities.

### Frequency and plans for auditing trial conduct {23}

Qualified trial staff will review Case Report Form (CRF) data for errors and missing key data points.

#### Direct access to participant records

Participating investigators must agree to allow trial-related monitoring, including audits, REC review, and regulatory inspections, by providing access to source data and other trial-related documentation as required. Participant consent for this must be obtained as part of the informed consent process for the trial.

#### Trial oversight

Trial oversight is intended to preserve the integrity of the trial by independently verifying a variety of processes and prompting corrective action where necessary. The processes reviewed relate to participant enrolment, consent, eligibility, and allocation to trial groups; adherence to trial interventions and policies to protect participants, including reporting of harms; completeness, accuracy, and timeliness of data collection; and will verify adherence to applicable policies detailed in the Compliance section of the protocol.

### Plans for communicating important protocol amendments to relevant parties (e.g. trial participants, ethical committees) {25}

Protocol amendments will be agreed upon between the co-investigators and Trial Steering Committee. They will be submitted by the PI to the Research and Ethics Committee and disseminated to the study team.

### Dissemination plans {31a}

#### Trial results

The results of the trial will be published in peer-reviewed journals and may be presented at academic conferences. The results will be communicated to trial participants who have requested to be informed. Results will be reported within 6 months of the end of the trial. The results of the trial will be disseminated regardless of the direction of effect.

Publication and dissemination of the results will be coordinated by the FANTAZE trial team, following the UCL Publication Policy.

## Discussion

Two recent pre-print papers [9, 10] on the viral dynamics of SARS-CoV-2, the cause of COVID-19, provide simulations to show that viral load peaks around 3 days after symptom onset and that antiviral therapy started later than day 4 may have limited benefit. Furthermore, late antiviral therapy, once viral loads are high, risks selection for drug resistance. Therefore, there are strong grounds to believe that if antivirals are to have a place in the treatment of SARS-CoV-2, they should be given early and, if possible, within the first 7 days of symptom onset.

Both recent SARS-CoV-2 modelling papers [9, 10] suggest that drugs need to provide a minimum of 65% inhibition of viral replication, with this percentage increasing to 90% or more if therapy is initiated late, meaning a combination therapy may improve the chances of achieving sufficient viral inhibition.

Favipiravir is orally administered and well absorbed with close to 100% bioavailability [11] . The drug is metabolized to a ribosyl triphosphate form which specifically inhibits the RNA-dependent RNA polymerase without affecting host RNA and DNA polymerases, thereby rendering it a specific inhibitor of viral RNA synthesis. The highest dose used in Phase II trials, for which safety is established, and hence proposed in this trial is 1800 mg twice daily on day 1 followed by 800 mg twice daily.

Favipiravir has been used clinically in several viral infections and in some cases, the drug has been observed to induce viral mutagenesis [12]. Occasional asymptomatic elevation of liver aminotransferase levels was observed, but no serious adverse events related to the drug. Importantly, animal studies have indicated potential teratogenicity and the drug is distributed in sperm: therefore, a negative pregnancy test for all women of child-bearing potential will be required and participants instructed to use adequate contraception for the duration of the treatment and 7 days afterward [13].

As stated above, favipiravir has been used to treat patients with COVID-19 in two Chinese studies [1, 2]. The first study [1] reported that favipiravir was administered (open-label) to 35 patients with COVID-19 and the outcome was compared to 45 patients treated with lopinavir/ritonavir in the preceding week; all patients also received inhaled interferon and supportive care. The median time to viral clearance in the favipiravir-treated group was 4 days compared to 11 days in those who did not receive favipiravir (*p*<0.001). At Day 14 from the start of treatment, chest CT scans had improved in 91.4% of favipiravir-treated patients compared to 62.2% of those who did not receive favipiravir (*p*=0.004).

In the second study [2] patients were consecutively recruited and randomly assigned to receive favipiravir or umifenovir (arbidol), a drug used for influenza; 120 participants were enrolled in each arm. In participants without severe disease, clinical recovery at 7 days was higher in the patients who received favipiravir (71.4% vs 55.9%, *p*=0.02). Time to resolution of fever and cough was also quicker in the favipiravir-treated group without severe illness. However, there was no significant difference in time to recovery among patients with severe disease, supporting the hypothesis that antiviral agents are best administered early in the disease course and certainly before the onset of respiratory failure.

Favipiravir was also used for Ebola virus disease, especially in the JIKI trial in Guinea [14]. Doses (2400mg/2400mg/1200mg on Day 1 followed by 1200mg twice daily) were significantly higher than proposed for the current study. No drug-related grade 3 or 4 clinical events were observed.

On the other hand, several studies involving the administration of nitazoxanide either alone or in combination to treat SARS-CoV-2 infections at different evolution states are registered at ClinicalTrials.gov (NCT04486313, NCT04361318, NCT04348409, NCT04523090, NCT04359680, NCT04552483, NCT04788407, NCT04561219, NCT04498936, NCT04382846, NCT04605588, NCT04563208, NCT04746183); however, no conclusive results have been reported up to the date of the registration of this trial.

Following oral administration, nitazoxanide is rapidly absorbed and then hydrolysed by plasma esterases to form tizoxanide. Tizoxanide is the active metabolite that is responsible for antiviral activity, and it is cleared from the body mainly by glucuronidation. Following oral administration of nitazoxanide, maximum plasma concentrations of tizoxanide and tizoxanide glucuronide are observed within 1–4 h and bioavailability is increased if the drug is taken with food. The parent nitazoxanide is not detected in plasma.

The proposed dose design for this trial is based on a study recently published by the University of Liverpool [15]. The study concluded that nitazoxanide concentrations in both plasma and lung can be achieved within the safe range of the drug and at the same time exceed the EC90 for SARS-CoV-2. The reported model and dosing strategies provide justification for the design of the FANTAZE clinical trial dosing regimen. These investigators constructed a PBPK model for oral administration of nitazoxanide which they validated against pharmacokinetic data in healthy volunteers. These volunteers had received doses between 500 and 4000 mg with and without food. This validated model was used to predict the dose with which plasma and lung concentrations could be achieved and maintained above the EC90 reported for nitazoxanide in more than 90% of the simulated population.

The combination of favipiravir and nitazoxanide has been included in a trial in South Africa as one of the combinations of a phase 2, exploratory, randomized, single-centre, open-label study of four different experimental treatment arms versus standard of care for the treatment of SARS-CoV-2 infection in symptomatic outpatients with mild disease at the time of enrollment [16]. In this study, one group of patients received: paracetamol, 1000 mg to be taken 6-hourly as needed; favipiravir, 1600 mg 12-hourly for 1 day then 600 mg 12-hourly for 6 days; nitazoxanide, 2 tablets (1000 mg) 12-hourly for 7 days. This trial completed recruitment in Sep 2021 but, to our knowledge, results have not been published.

The preliminary results seen for favipiravir, and the promising in vitro activity of nitazoxanide, urgently need to be confirmed (or refuted) in a well-conducted, placebo-controlled trial. Therefore, there is a strong rationale for choosing to trial early oral antiviral therapy with favipiravir and to investigate its combination with nitazoxanide.

## Trial status

The protocol version 1.2 was approved by the National Committee of Scientific Investigation (IRB00003566) on Feb 10, 2021; and by the Federal Commission for the Protection against Sanitary Risks (COFEPRIS) on March 19, 2021. The protocol version 1.3 was approved by the National Committee of Scientific Investigation (IRB00003566) on July 21, 2021. The current version of the protocol is 1.4, dated November 12, 2021, approved by the National Committee of Scientific Investigation (IRB00003566) on Dec 4, 2021. Recruitment began on Nov 18th, 2021, and is expected to be completed by August 30, 2022.

## Data Availability

The PI, CPM, Trial Manager, Data Manager, Statistician, and Trial Management Team will have full access to the trial data. Requests for access to trial data will be considered, and approved in writing where appropriate, after formal application to the TSC. The results of the trial will be published in peer-reviewed journals and may be presented at academic conferences. The results will be communicated to trial participants who have requested to be informed. Results will be reported within 6 months of the end of the trial. The results of the trial will be disseminated regardless of the direction of effect. Publication and dissemination of the results will be coordinated by the FANTAZE trial team, following the UCL Publication Policy.

## Abbreviations

AE: Adverse event
ALT: Alanine aminotransferase
AE: Adverse reaction
AST: Aspartate aminotransferase
BID: Two times a day
CCTU: Comprehensive Clinical Trials Unit at UCL
CINVESTAV: Investigation and Advanced Studies Institute Of The National Polytechnical Institute
COFEPRIS: Federal Commission For The Protection Against Sanitary Risks
CRF: Case Report Form
DSUR: Development Safety Update Report
EC: Ethics Committee
EC50: Half Maximal Effective Concentration
eGFR: Estimated glomerular filtration
IB: Investigator’s Brochure
ICF: Informed consent form
ICH-UCL: UCL Great Orment Street Institute of Child Health
ICU: Intensive care unit
IDMC: Independent Data Monitoring Committee
IMSS: Mexican Social Security Institute
IMP: Investigational Medicinal Product
ITT: Intention-to-treat
LVP/r: Lopinavir plus ritonavir
PBPK: Physiologically based pharmacokinetic model
PD: Pharmacodynamics
PI: Principal Investigator
PK: Pharmacokinetics
PPE: Personal protective equipment
QID: Four times a day
R&D: Research and development
SAE: Serious adverse event
SAP: Statistical analysis plan
SPC: Summary Of Product Characteristics
SUSAR: Suspected unexpected serious adverse reaction
TID: Three times a day
TMG: Trial Management Group
TSC: Trial Steering Committee
UCL: University College London
ULN: Upper limit of normal
